# Economics of Philanthropy

**Published:** 1898-06-11

**Authors:** 


### THE INSTITUTIONAL WORKSHOP.

### ECONOMICS OF PHILANTHROPY.

IX.

The admitted difficulty in questions of philanthropy is that while there are many cases which ought to be treated by private benevolence rather than by legal relief, there is so much danger of admitting to this latter aid persons who will take undue advantage of it. The public mind is now so much awakened to the folly of encouraging mendicity that it is doubtful if the large incomes which beggars were accredited with making are now to be had. We don't think that "Miss Shum's husband," if he were alive to day, would be able to live luxuriously on the profits of crossing-sweeping. But there are certainly many ingenious persons who devote very considerable energy to the task of getting a donation from this society and an annuity from that, food tickets, medicine, surgical appliances, everything that they can use or sell. To restrain these robbers of the charitable, we have societies which devote themselves to inquiring into the claims of all applicants. So far as the repression of mendicity goes these societies do their work very well. But the whole duty of charity is not to withhold, though that may often be necessary; but also, when it is wise, to give. Now, giving cannot be done by machinery; and societies often tend to become more or less machines. Hard and fast forms of application and investigation may—but do not inevitably—restrain the unworthy from asking help; but they do, much more frequently, keep the worthy from doing so. We have seen a rather profane but very brilliant skit on the methods of one society, which shows that if one of its officers had filled up the regulation form regarding Jesus of Nazareth, the Son of God would have been refused help as a tramp and a beggar. Allowing for the fact that the conditions of life and charity are different in modern England from what they were in ancient Judæa, we are nevertheless forced to admit that many cases which would not, when set down on a given form, which tells much but omits more, command help, are yet deserving of sympathy and assistance. Two difficulties arise in dealing with things thus. In the first place, sensitive persons, however needy, will not submit to this inquisition, conducted, as it often is, without regard to that privacy to which even the poorest has a right; and, in the second place, it leaves no opening for those numerous cases in which the help required is not alms, but advice.

The poor are weak in knowledge of the world. They know the history of the street and the geography of the slum, but there their information ends. A man, say, is out of work. For reasons quite beyond his ken, the industry by which he has hitherto lived is leaving the neighbourhood. It may not have gone very far away; but he does not know this—does not know where to go in search of it. What he wants in such a case is not alms to keep him and his family a longer or shorter time out of the workhouse, but the information where to go, and perhaps the loan of money enough to take him thither. It is very difficult for the middle and upper classes, who are from their childhood accustomed to hear of the doings of every part of the world, who have friends in every quarter of the globe, to realise how limited is the mental horizon of their poorer neighbours. Equally difficult is it for them to understand how little the poor know of business matters, which "come by nature" to themselves. All that travel, intercourse with society, wide and miscellaneous reading teach us is unknown to the poor; and our educational system, though it may put the means of acquiring information in their way, cannot show them how to acquire it. Everyone who has gone much among the poor must have been surprised at the ignorance of things outside a very narrow circle which men and women show, who within that circle are remarkably shrewd. To extend their knowledge of the world is to extend their chances of getting on to an almost incalculable amount. It is for this reason that societies, though managed on the severest principles, often fail to lift their protégés permanently out of the ranks of poverty. They may relieve their immediate wants, but they give them but little guidance as to the conduct of their future. For this a personal acquaintance with the special circumstances of the case is necessary, such as no rules can supply, and which no one individual can give to a whole army of cases. It is better for a man who loves his fellow-men to devote all the time and attention he can spare to the amelioration of one or two poor families than to give a casual and inefficient sympathy, however accompanied it may be with alms, to a number of persons whom he cannot intimately know. It is very often possible for one man who has the advantages of education, business training, and money to help another who has little or none of these things; he can impart them in such degree as they are required; but it is almost impossible for him to give efficient help to many. The mass of misery around us distresses and bewilders us, and in our aimless attempt to assist all we often fail to be of help to any single one. The best thing that any association for regulating charity can do is to bring the one who needs help into contact with one who can help him. Of course, this implies, on the part of the helper, a readiness to give time—time rather, and in greater measure, than money. The pity of it is that with those who could be most helpful time is so often more precious than money that they cannot afford it.

But while individual care and work is necessary, it is better that the individual helpers should be associated in such a degree that they do not interfere with each other's work, otherwise we are back at the old bad point, where the unscrupulous poor prey upon those who seek to help them. There must be free and full intercourse between all kinds of charitable workers. The best plan we have heard of as being carried out on a large scale is that of the Bureau of Associated Charities of Boston. Before it all cases come, those which are suitable only for public relief as well as those that are fitted for the gentler means of private charity. The knowledge that those who are dealt with in the bureau may be sent to the poorhouse acts as a deterrent to the unworthy, while the knowledge that they will not inevitably be forced to accept a form of relief which they consider degrading because they go to the bureau, makes it easier for deserving cases to bring their needs forward. Then from this centre every form of help can radiate, and suitable assistance given to every case. The variety of helpers makes it easier than it would otherwise be to find work for those who are fit for it, and to place all according to their necessities and their deservings. Help is increased, and at the same time waste is minimised. In this country, though it is within the power of a charitable association to refuse help, and refer an applicant to the poor-law authorities, it is not a usual thing for the latter to send an applicant to a private charity; that is not within their province. Yet the one thing might be as possible as the other, and the really needy who hardly know where to go for help, other than alms, the help that would be permanently useful, should be able to find it through the one avenue they know of—the poor law. Without such reciprocity, we shall always have a section of the poor who are suffering bitterly, because they will not accept poor-law relief, and do not know where efficient and non-humiliating assistance is to be found; and, on the other hand, a section of the philanthropic public who will lavish unnecessary and undeserved sympathy upon those who will not accept poor-law relief, whether their refusal be founded upon a dread of the enforced cleanliness and sobriety of the workhouse, or a genuine self-respect which hates the idea of dependence. Till some such working together between public and private charity is made general it is hardly possible to make the work of philanthropy either as efficient or as economical as it ought to be.

